# Advances in Thyroid Carcinoma

**DOI:** 10.3390/cancers14122908

**Published:** 2022-06-13

**Authors:** Iñigo Landa

**Affiliations:** Division of Endocrinology, Diabetes and Hypertension, Department of Medicine, Brigham and Women’s Hospital & Harvard Medical School, Boston, MA 02115, USA; ilanda@bwh.harvard.edu

“Thyroid cancer” encompasses a heterogeneous group of tumors that range from the predominant papillary thyroid cancer (PTC) subtype, which shows excellent survival rates, to the poorly differentiated (PDTC) and anaplastic thyroid cancer (ATC) forms, accounting for most of the disease-related morbidity and mortality. The term also refers to tumors arising from parafollicular or C cells, resulting in medullary thyroid cancers (MTC) [1]. The last decade has witnessed a dramatic improvement in our understanding of the genomic and biological underpinnings of these tumors, which, in some cases, have been translated into the clinic, e.g., to refine diagnostic and prognostic, or to inform patient stratification for targeted therapies. Despite this remarkable progress, some challenges remain. To name a few, from a research perspective, we still do not fully understand which specific genetic lesions, and more importantly, which underlying mechanisms govern cancer biology in a subset of thyroid tumors. On the clinical front, ATCs remain almost invariably fatal cancers, and patients treated with targeted therapies eventually develop resistances. In this Special Issue entitled “Advances in Thyroid Carcinoma”, we present a collection of 18 articles, including 13 research papers and 5 reviews, from groups across the globe, addressing some of these topics.

Our Special Issue includes several papers reporting on the genomic features of various thyroid cancer patient cohorts. Jin et al. evaluated 36 tumors from aggressive PTC subtypes, i.e., tall-cell and columnar-cell variants, for the presence of mutations in genes that had been previously shown to be enriched in PDTCs and ATCs [2]. They found that the frequency of these lesions typically lies between that of the most indolent PTCs (profiled by The Cancer Genome Atlas (TCGA)) and advanced thyroid tumors (PDTC/ATC), supporting the idea of a continuum in thyroid cancer evolution [3,4]. Similarly, Póvoa, Teixeira and colleagues reported a single-institution series of 241 PTC patients who underwent surgery and showed that *TERT*-promoter mutations, alone or in combination with BRAF^V600E^, are associated with an increased risk of developing structural disease and disease-specific mortality [5], thus providing further evidence of *TERT* as molecular marker of aggressivity [6]. Liu et al. took an alternative approach and analyzed publicly available DNA methylation data from TCGA-profiled thyroid cancers, defining a subset of tumors with a so-called “highly drifted DNA methylation age” that tend to correlate with lower differentiation scores and might serve as a marker of the activation of certain pathways and of specific tumor characteristics [7]. Misiak and colleagues unveiled the diagnostic potential of microRNA (miRNA) deregulation in ATC, and, by profiling messenger RNA (mRNA) expression in the same specimens, their consequences for differential gene expression. Notably, the authors were able to identify inverse correlations between up- or downregulated miRNAs and the concomitant down- and upregulation of their targets, e.g., *FRMD3* (a putative tumor suppressor role in ATCs) and *BIRC5* (oncogene), respectively [8]. Carneiro et al. performed the RNA-sequencing of thyroid tumors driven by *RAS* mutations, reporting that missense mutations and expression changes in genes belonging to the Hippo pathway accumulate in these specimens, suggesting that they cooperate with the RAS oncoprotein in thyroid tumorigenesis [9]. Finally, Minna and colleagues characterized the mutational and transcriptomic characteristics of a cohort of MTCs, and performed a meta-analysis of other published studies, delineating other potential driver and signaling properties that might help to refine the classification of this tumor type [10].

Two studies of this Special Issue focused on the genetic characteristics of PTCs with particularly challenging features from a treatment standpoint. Siraj and colleagues employed whole-exome sequencing (WES) to show that *TERT*-promoter mutations and the APOBEC SBS13 mutational signature are both independent predictors of refractoriness to radioiodine (RAI) treatments in PTCs (compared to their RAI-avid counterparts) [11]. Bagheri-Yarmand, Busaidy and colleagues characterized a metastatic PTC case that developed an acquired resistance to the BRAF inhibitor dabrafenib, demonstrating that this resistance is mediated, at least in part, by acquired alterations at *RAC1* gene (encoding a GTPase), and perhaps the co-amplification of other genes within chromosome 7. The authors showed, by employing patient-derived cell lines and isogenic cell models, that *RAC1* alterations enhance growth, invasion and signaling via phospho-PAK1, suggesting potential vulnerabilities for combinatorial therapies [12].

This collection of papers also encompasses various studies assessing the specific mechanisms of thyroid cancer progression. Caperton, Jolly et al. described the establishment of three follicular thyroid cancer (FTC) cell lines derived from murine models driven by oncogenic Hras^G12V^ and *Pten* loss. The authors showed that these cell lines are suitable models to explore MAPK- and PI3K-pathway-related therapeutic dependencies, and that they can be used as syngeneic models of progression to PDTC/ATC upon subcutaneous injection into mouse hosts [13]. Stempin, Geysels and colleagues dissected the functional role of tumor-associated macrophages (TAMs) in ATC. The authors demonstrated that ATC-cell-derived conditioned media induces the M2 polarization of human monocytes in vitro, and that this process is mediated by TIM3, an immune regulatory molecule whose blockade could be a putative therapeutic strategy for ATC treatment [14]. Thornton, Hao et al. evaluated the regulation of *TERT* mutant promoter by the ETS family of transcription factors, showing that, compared to other lineages, thyroid cancers show a more intricate, yet not fully characterized, control of *TERT*-mutant transcription, which is likely mediated by multiple ETS proteins (beyond the reported GABPA) as well as MAPK pathway inputs [15].

This issue comprises two clinical research papers in patient cohorts with long-term follow-up data. Siraj et al. reported a large PTC cohort and observed a two-peak pattern of recurrence for intermediate- and high-risk tumors, which might inform refined strategies for patient surveillance [16]. Steinschneider and colleagues followed up differentiated thyroid cancer (DTC) patients with a biochemical incomplete response (BIR), showing that, although many BIR patients achieve the “no evidence of disease” status, some display structural recurrences, suggesting that a more individualized approach should be considered for the latter group [17].

This Special Issue also features interesting review articles. To commemorate the 80th anniversary of the use of radioiodine therapy, De la Vieja and Riesco-Eizaguirre provided an in-depth review of the molecular and clinical considerations of the use of radioiodine treatment, as well as aspects on the sodium/iodide symporter (NIS) biology, in thyroid cancers and other diseases [18]. Fukuda and Takahashi, on their part, reviewed the clinical factors that can be helpful in determining the use of multi-kinase inhibitors in patients with RAI-refractory DTC, with a focus on clinical trials employing sorafenib or lenvatinib [19]. Wijewardene and colleagues summarized the evidence supporting the use of liquid biopsies, including the study of both circulating tumor cells and circulating tumor nucleic acids (in blood), for the diagnosis and prognosis of thyroid cancer patients [20]. Fröhlich and Wahl reflected on the potential future clinical application of nanoparticles for the diagnosis and delivery of treatments in aggressive thyroid tumors, such as ATC [21]. Fuziwara et al. provided a comprehensive overview of gene-editing methods via CRISPR/Cas9 technologies, including a summary on how these approaches have been applied to modify genes involved in the pathogenesis of thyroid cancers [22].

In summary, this Special Issue of *Cancers* includes a collection of articles covering topics on basic, translational and clinical studies in thyroid carcinomas that will be of interest for a wide audience of researchers and clinicians working on these tumors.
